# The vomeronasal system of the newborn capybara: a morphological and immunohistochemical study

**DOI:** 10.1038/s41598-020-69994-w

**Published:** 2020-08-06

**Authors:** Mateo V. Torres, Irene Ortiz-Leal, Paula R. Villamayor, Andrea Ferreiro, José Luis Rois, Pablo Sanchez-Quinteiro

**Affiliations:** 1 Department of Anatomy, Animal Production, and Clinical Veterinary Sciences. Faculty of Veterinary, University of Santiago de Compostela, Av Carballo Calero s/n, 27002 Lugo, Spain; 2 Marcelle Nature Park, Outeiro de Rei, Lugo, Spain

**Keywords:** Development of the nervous system, Olfactory system, Anatomy, Nervous system, Immunohistochemistry, Microscopy

## Abstract

The vomeronasal system (VNS) is responsible for the perception mainly of pheromones and kairomones. Primarily studied in laboratory rodents, it plays a crucial role in their socio-sexual behaviour. As a wild rodent, the capybara offers a more objective and representative perspective to understand the significance of the system in the Rodentia, avoiding the risk of extrapolating from laboratory rodent strains, exposed to high levels of artificial selection pressure. We have studied the main morphological and immunohistochemical features of the capybara vomeronasal organ (VNO) and accessory olfactory bulb (AOB). The study was done in newborn individuals to investigate the maturity of the system at this early stage. We used techniques such as histological stains, lectins-labelling and immunohistochemical characterization of a range of proteins, including G proteins (Gαi2, Gαo) and olfactory marking protein. As a result, we conclude that the VNS of the capybara at birth is capable of establishing the same function as that of the adult, and that it presents unique features as the high degree of differentiation of the AOB and the active cellular migration in the vomeronasal epithelium. All together makes the capybara a promising model for the study of chemical communication in the first days of life.

## Introduction

The vomeronasal system (VNS) is the sensorial system responsible in most vertebrates for the detection of chemosensory signals linked to innate socio-sexual behaviours^1,2^. In mammals, the VNS presents a high morphofunctional^3^ and genomic^4^ diversity among different species. The vomeronasal organ (VNO) specialises in detecting pheromones for the purpose of reproductive behaviours such as maternal aggression and sexual attraction^5^. The VNS is also involved in the recognition of major histocompatibility complex (MHC) associated peptides^6^, kairomones^7^ and aversive molecules^8^.

By performing an in-depth study of the macroscopic and microscopic morphological characteristics of the vomeronasal system in the newborn capybara, we aimed to achieve two objectives. On the one hand, we aimed to obtain general information regarding the vomeronasal system in a rodent model that is distinct from most studied laboratory rodents. On the other hand, because the capybara is a precocial animal species, we aimed to determine the degree to which the capybara vomeronasal system morphology at birth has adapted to the requirements of a demanding socio-cognitive environment.

Most studies of the VNS have been done on laboratory rodent strains, exposed to artificial selection pressure that do not reflect the selection pressure present in the wild. Therefore, these laboratory strains present significant genetic and behavioural differences compared with wild rodent models^9^. The laboratory mouse (*Mus musculus*) and rat (*Rattus norvegicus*) may not be representative of all animals that make up this family.

A remarkable differential feature among rodents is the altricial character of mice and rats, compared with the precocial character presented by hystricomorphic rodents, such as the guinea pig or capybara^10^. Altricial neonates lack fully developed senses and require extensive parental care, such as brooding or food provisioning. In contrast, precocial species are born with more developed senses, require limited parental care, and can feed self-sufficiently, early on, while still being nursed^11^. Differences in the maturation of sensory systems between altricial and precocial species may provide insight regarding behavioral development patterns, in both groups.

Both the main olfactory and vomeronasal systems act synergistically and constantly interact during development^12^. Although the age at which the main olfactory system acquires adult-like morphology has been well-established to demonstrate a more-organized system at an earlier postnatal age among precocial species compared with altricial rodents^13,14^, less information is available regarding the VNS during the early postnatal development of different rodent species. The development of the VNS has been exclusively assessed in immature altricial species, including the mouse and rat^15^,however, no studies have examined the morphological and functional maturity of the VNS in precocial rodents during the perinatal period.

Hystricognathi has become a model group for the study of the anatomical diversity of the VNS, as shown by the studies in chinchillas^16,17^, guinea pigs^18^, degus^19,20^, and mole rats^21,22^. Suárez et al.^23^ paid special attention to the organisation of the first integrative centre of the VNS, the accessory olfactory bulb (AOB) in capybaras *Hydrochoerus hydrochaeris*, particularly to the morphometry of the anteroposterior zonation, which is determined by the expression of the G proteins. These authors showed how the Gαo-positive AOB caudal subdomain in capybaras is larger than the rostral subdomain, which differs from the *Octodon degus* AOB, which presents a larger Gαi2 anterior region. Whereas capybaras are semi-aquatic mammals, whose chemocommunication relies mostly on the oily secretions associated with male-to-male pheromonal communications, the degus lives in semiarid spaces and prevalently establishes male–female interactions. Therefore, this study suggests that ecological specialisations may play important roles in shaping the AOB.

The present study describes the anatomy, histology, and histochemical and immunohistochemical features of the VNS of the newborn capybara and discusses its functional status at birth. We employed dissection, microdissection, histological staining and immunohistochemical techniques. Three lectins were studied: *Ulex europaeus* agglutinin (UEA), specific for the canid vomeronasal system^24^,*Bandeiraea simplicifolia* isolectin B_4_ (BSI-B_4_), which marks the VNS in both rats^25^ and opossums^26^,and *Lycopersicon esculentum* agglutinin (LEA), a specific marker for both olfactory systems.

The immunohistochemical study covered a large number of antibodies, which provides useful information on VNS function. Antibodies against Gαi2 and Gαo, were used to determine which pheromone receptor families—V1R^27^ or V2R^28^, respectively—are expressed in the VNS. The mitral cells, primary neural elements of the AOB, were labelled with antibodies against microtubule-associated protein 2 (MAP-2). The neuronal growth, especially important during the first stages of life, was studied by employing anti-growth-associated protein 43 (GAP-43) and anti-Luteinizing hormone-releasing hormone (LHRH). The maturity of the system was determined using anti-olfactory marker protein (OMP). The calcium-binding proteins calbindin (CB) and calretinin (CR) were used to identify neuroactive substances. Astrocytes and ensheathing cells were recognised by an antibody against glial fibrillary acidic protein (GFAP).

Our study aimed to address current gaps in our understanding of the rodent vomeronasal development, through ontogeny, by providing essential information regarding the newborn capybara VNS, showing that this species presents an advanced stage of structural maturity during the first days of life. The macroscopic, histological and immunohistochemical peculiarities and differences from the VNS of mice and rats demonstrate the wide diversity of the VNS between even closely related species, supporting the necessity of studying each species individually to avoid making incorrect extrapolations.

## Material and methods

Through a collaboration with Marcelle Nature Park (Outeiro de Rei, Spain), we were provided with three one-day-old capybaras (*Hydrochoerus hydrochaeris*) for use in this study. The animals died by perinatal causes, and they were two males and one female.

The heads were separated and introduced into the fixative after removing the jaws and extracting the skin, muscular plane and other structures such as the tongue and eyes. A window was opened dorsally in the skull in the proximity of the olfactory bulbs to facilitate the penetration of the fixative. The fixatives used were 10% formol and freshly prepared Bouin’s fixative. The latter is especially suitable for the study of the nervous system due to its superior penetration capacity and because it lends consistency to the tissues, thus facilitating its subsequent processing. After 24 h, the samples were transferred into 70% ethanol.

### Sample extraction

We focused the extraction of the samples on the following anatomical structures: the nasal cavity (NC), vomeronasal organ (VNO), vomeronasal nerves, and olfactory bulbs (OBs).

*Nasal cavity* The entire NC was separated by a transverse incision made rostrally to the ethmoidal fossa to prevent damage to the olfactory bulbs. The resulting sample was used to study the macroscopic and microscopic changes in the topography of the VNO throughout the NC.

*Vomeronasal organ and nerves* After opening the NC using a rotating saw, the dorsal and ventral turbinates were removed. This allowed the visualisation of the nasal septum in its entirety, over which the vomeronasal nerves were dissected. Once the VNOs were identified on both sides of the base of the anterior portion of the nasal septum—and because of their small size and the close contact they have with the vomer bone—it was necessary to extract them with the help of a surgical microscope (Zeiss OPMI 1 Ent).

*Main and accessory olfactory bulbs* The complete removal of the cranial vault was performed using a gouge forceps. It was begun caudally to take advantage of the lower resistance presented by the bone at this level. Special care was taken when approaching the OBs, located deep in the ethmoidal fossa, since they are extremely delicate. To access the OBs, the bony orbital fossa, which laterally covers the bulbs was removed. Finally, using a scalpel, the dura mater and the olfactory nerves were dissected together since both structures hold the bulbs against the ethmoidal cribriform plate.

### Sample processing for histological study

Paraffin embedding was used to perform the histological processing of all samples (VNOs and OBs). In one of the individuals, the complete NC was pre-decalcified; it was immersed in a decalcifying solution (Shandon TBD-1 Decalcifier, Thermo, Pittsburgh, PA, USA) and continuously stirred for thirty hours. The samples were then washed under running water for two hours, and were cut into several blocks which were serially cut from the incisor papilla to the caudal end of the vomeronasal cartilage in order to obtain information on the changes in the VNO throughout its length. Following this process, all blocks were paraffin embedded.

*Cutting* The samples were cut with a Leica Reichert Jung microtome with a thickness of 4–8 μm, depending on the tissue to be processed. We opted for thinner cuts in the study of the VNO and thicker cuts in the study of the AOB, as these allow a better visualisation of the nerve and glial processes.

### General histological staining

In order to highlight the different tissue components, we used the following stainings: Haematoxylin–Eosin (HE) as a general staining, periodic acid-Schiff (PAS) and Alcian Blue (AB) for neutral and acid mucopolysaccharides, respectively, and Nissl staining for the brain tissue. Additionally, two specific stainings, Gallego’s Trichrome and Tolivia, were performed.

*Gallego’s trichrome* This stain allows for the differentiation of components of the connective tissue. In non-decalcified samples, it stains erythrocytes green, muscle fibres and collagen light blue, epithelium and glandular tissue red, bone dark blue and cartilage purple. In decalcified samples, the pattern varies slightly; the glandular tissue appears light blue, the muscle fibres and the epithelia are stained green^29^. The protocol used was as follows: sections were deparaffinised and rehydrated to stain with Ziehl acetic fuchsin for 2 min (10 drops Ziehl fuchsin, 1 drop acetic acid, 10 cc distilled water). After washing them with distilled water, they were introduced into formalin–acetic acid solution for 5 min (2 drops formalin, 2 drops glacial acetic acid, 10 cc distilled water). After two more washes, the sections were finally introduced into picroindigocarmine for 3–5 min (one part 1% indigocarmine aqueous solution, two parts saturated aqueous picric acid solution).

*Tolivia* This technique stains the myelinated nerve fibres a black colour and the neuronal somas a pink colour. To favour the fixing of the dyes, the sections were introduced into 2.5% FeNH_4_(SO4)_2_ for 1 h. The myelin stain solution was prepared freshly as follows: 5 ml of 20% haematoxylin plus 10 ml of 1% Li_2_CO_3_ in 50 ml of 50% ethanol. The samples were in this solution for 2.5 h. After three washes in tap water for 5 min, the slides were bathed for 5 min in a solution of 0.2% pyronine in 20% formaldehyde. Finally, the samples were dehydrated, cleared and mounted^30^.

### Histochemical and immunohistochemical staining

*Histochemical labelling (HQ) with lectins* This technique is based on the binding between certain proteins (lectins) and carbohydrates, which can be found free, in oligosaccharides or in glycoproteins. For our study, we used (1) a lectin that comes from the gorse, the *Ulex europaeus* agglutinin (UEA), which recognises α-L-fucose, (2) the α-galactose-specific BSI-B_4_ that comes from *Bandeiraea simplicifolia*, and (3) *Lycopersicon esculentum* agglutinin (LEA), a lectin coming from tomato with a high affinity for N-acetyl-β-D-glucosamine oligomers (Table 1). These stains selectively recognise the different components of the olfactory and vomeronasal pathways in some species. They have been used in both VNO and AOB sections.

**Table 1:** Antibodies and lectins used, with species of elaboration, dilution, manufacturer, and catalogue number.

Ab/Lectin*	1st Ab species/dilution	1st Ab Catalogue number	2nd Ab species/dilution (Catalogue number)
Anti-Gαo	Rabbit 1:100	MBL 551	ImmPRESS VR HRP Anti-Rabbit IgG Reagent MP-6401-15
Anti-Gαi2	Rabbit 1:100	Sta Cruz Biotechnology SC-7276	ImmPRESS VR HRP Anti-Rabbit IgG Reagent MP-6401-15
Anti-OMP	Goat 1:400	Wako S44-10001	Horse 1:250 Vector BA-9500
Anti-MAP2	Mouse 1:200	Sigma M4403	ImmPRESS VR HRP Anti-Mouse IgG Reagent MP-6402-15
Anti-GAP43	Mouse 1:800	Sigma G9264	ImmPRESS VR HRP Anti-Mouse IgG Reagent MP-6402-15
Anti-GFAP	Rabbit 1:400	Dako Z0334	ImmPRESS VR HRP Anti-Rabbit IgG Reagent MP-6401-15
Anti-Calbindin	Rabbit 1:5000	Swant CB38	ImmPRESS VR HRP Anti-Rabbit IgG Reagent MP-6401-15
Anti-Calretinin	Rabbit 1:5000	Swant 7697	ImmPRESS VR HRP Anti-Rabbit IgG Reagent MP-6401-15
Anti-LHRH	Rabbit 1:500	Fisher Scientific A235481	ImmPRESS VR HRP Anti-Rabbit IgG Reagent MP-6401-15
UEA-I*	1:10	Vector L-1060	Rabbit 1:50 DAKO P289
LEA*	20 µg/ml	Vector B-1175	Vectastain ABC reagent PK-4000
BSI-B_4_*	100 µg/ml	Sigma L-2140	Vectastain ABC reagent PK-4000

*Abbreviations* Gαo: Subunit αo of G protein; Gαi2: Subunit αi2 of G protein; OMP: olfactory marker protein; MAP-2: microtubule associated protein-2; GAP-43: growth-associated protein 43; GFAP: glial fibrillary acidic protein; CB: calbindin; CR: calretinin; LHRH: luteinizing hormone-releasing hormone; UEA: *Ulex europaeus* agglutinin; LEA: *Lycopersicum esculentum* agglutinin; BSI-B_4_: *Bandeiraea simplicifolia* isolectin B_4_; HRP: horseradish peroxidase; IgG: Immunoglobulin G; ABC: avidin–biotin-complex.

The protocol for the UEA is as follows. It begins by (i) blocking the endogenous peroxidase activity of the sample, avoiding possible interference with the developing solution. To do this, the sample is incubated in 3% H_2_O_2_ solution for 10 min and then (ii) incubated for 30 min in 2% bovine serum albumin (BSA), which prevents nonspecific binding. The next step is (iii) incubation with the UEA lectin for 1 h to visualise the lectin-carbohydrate junction followed by (iv) 3 × 5 min washes in 0.1 M phosphate buffer (PB, pH 7.2), and (v) incubating for 12 h in a peroxidase-conjugated immunoglobulin against the UEA. Finally, (vi) the sections were washed with PB and developed by (vii) incubation of the sections in a solution of 0.05% diaminobenzidine (DAB) and 0.003% H_2_O_2_ for 5 min.

The protocol for the LEA and BSI-B_4_ begins with the same two steps. Next, we (iii) incubated the sections overnight in biotinylated lectins diluted in 0.5% BSA. The next day, the samples were (iv) incubated for 1.5 h in Vectastain ABC reagent (Vector Laboratories, Burlingame, CA, USA). The samples were finally (v) developed by incubation in the same DAB solution as the UEA.

*Immunohistochemistry (IHQ) techniques* After deparaffinisation and rehydration, this protocol also began by (i) blocking the activity of endogenous peroxidase. Later, (ii) the non-specific binding was blocked with 2.5% horse normal serum from the ImmPRESS reagent kit Anti-mouse IgG/Anti-rabbit IgG (Vector Laboratories, CA, USA) for 30 min. (iii) The primary antibody was then added at the corresponding dilution (Table 1) and allowed to incubate overnight. The next day, (iv) the samples were incubated for 20 min with the corresponding ImmPRESS VR Polymer HRP Anti-Rabbit IgG Reagent. (v) After rinsing in Tris-buffer (pH 7.61) for 10 min, (vi) the samples were finally developed using DAB as a chromogen in the same way as for the lectins.

All immunohistochemical protocols were checked with the appropriate controls. In the absence of a positive control specific to capybaras, we replicated the entire histochemical procedure with mouse tissues known to express the proteins of interest. Samples for which the primary antibody was omitted were used as negative controls.

### Acquisition of images and digital treatment

Digital images were taken using the Karl Zeiss Axiocam MRc5 digital camera coupled to a Zeiss Axiophot microscope. Adobe Photoshop CS4 (Adobe Systems, San Jose, CA, USA) was used as needed to adjust parameters such as brightness or contrast, balance light levels, and crop or resize images for presentation in this work. Some photomicrographs were formed as a mosaic of several photographs merged with an image-stitching software (PTGui Pro, New House Internet Services BV, The Netherlands).

### Ethical approval

All the animals employed in this study dead by natural causes.

### Informed consent

No human subject was used in this study.

## Results

**Figure 1:**
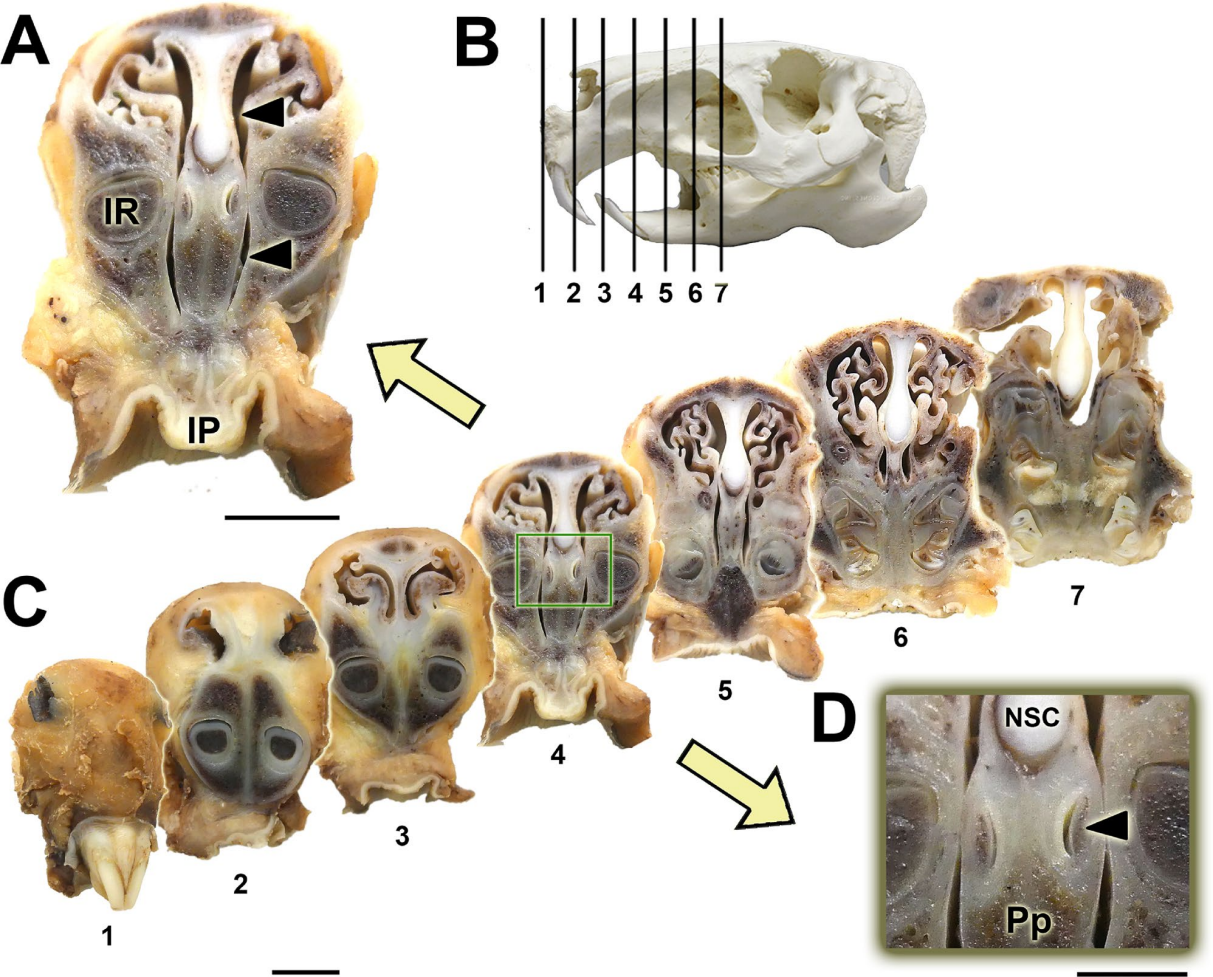
Cross sections of the nasal cavity in seven levels (**C**) ordered from rostral (1) to caudal (7). The levels of the sections are represented in the lateral view of the skull (**B**). The central part of the VNO is located at level 4 and can be seen at greater magnification in (**A**). In the same image, we can see the incisive papilla (IP) and the roots of the incisors (IR). At higher magnifications (**D**) two slits are observed that correspond to the vomeronasal ducts, associated with their cartilaginous capsule (arrow). Both organs are located in the central part of the nasal septum, dorsal to the incisive bone (Pp) and ventral to the cartilage of the nasal septum (NSC). Scale bars: (**A**,**C**) 1 cm; (**D**) 0.5 cm.

**Figure 2:**
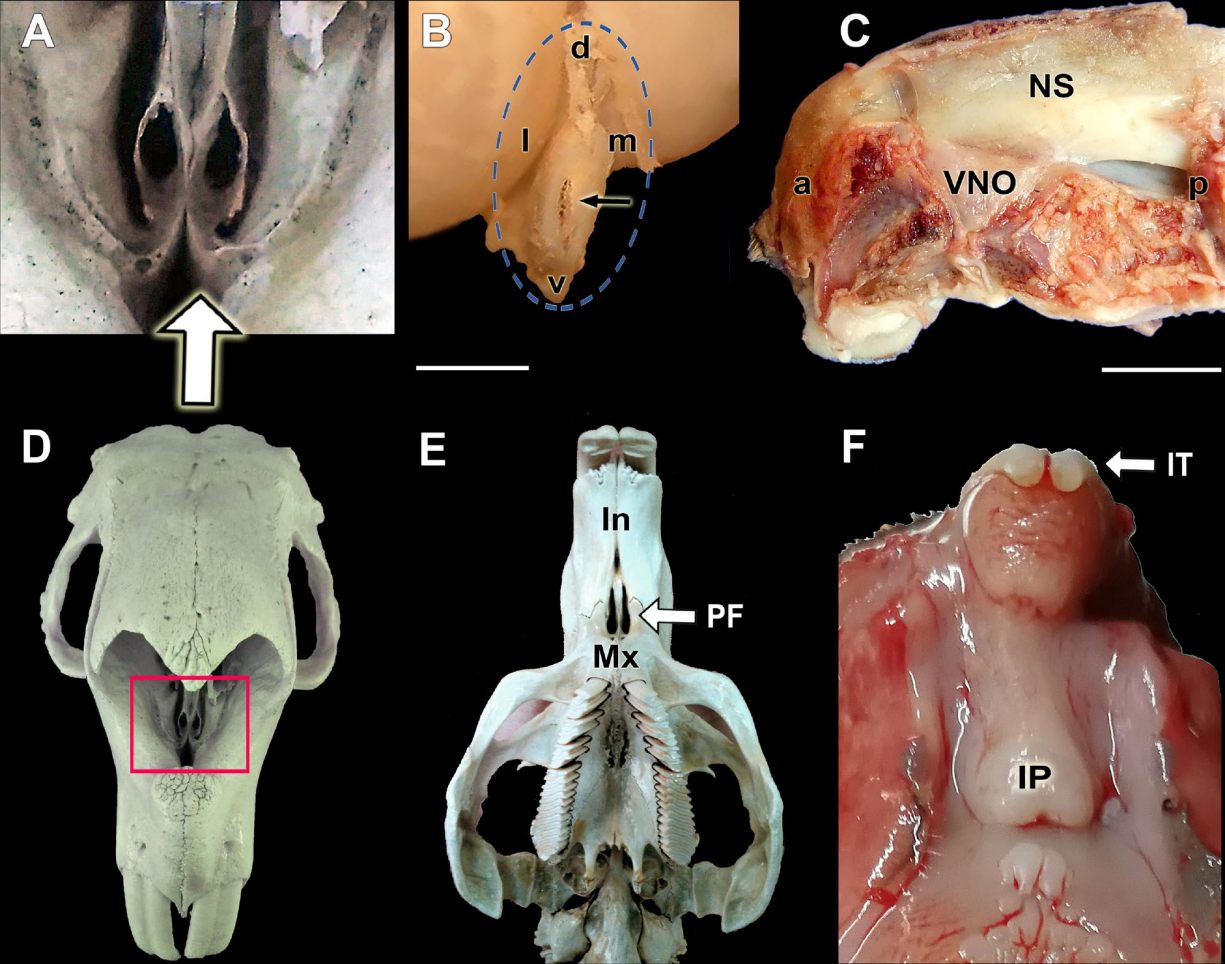
Dissection of the VNO and the incisive papilla. (**A**–**D**) The adult capybara skull gives us the first information on the topographic features of the VNO. (**D**) The dorsorostral view of the skull shows the bony structures that support the caudal third of both organs. The inset is shown in (**A**). (**B**) VNO cross section after its extraction, bounded by the blue circle. Arrow: VNO capsule. (**C**) Dissection of the deep plane of the left nasal cavity. The VNO corresponds to the triangular area in the anteroventral part of the nasal cavity. (**E**) Ventral view of an adult capybara skull showing the palatine fissures (PF). (**F**) Roof of the oral cavity of the neonate capybara showing the incisive papilla (IP). a: Anterior, p: posterior, d: dorsal, l: lateral, m: medial, v: ventral; In: Incisive bone; IT: Incisor teeth; Mx: Maxillary bone; NS: Nasal septum. Scale bars: (**B**) 2 mm; (**C**) 1 cm.

The main macroscopic features of the capybara VNO are depicted in Figs. 1,2. A series of cross sections were made along the previously decalcified nasal cavity (Fig. 1). By this method, we identified the organ located in the central levels where the cavity is T-shaped and dorsally occupied by the turbinates (Fig. 1A). Ventrally, a recess, formed by the presence of the roots of the incisors, is occupied by the prominent dorsal projection of the palatine process of the incisive bone (Pp), on which both VNOs rest.

The VNO was dissected in the other two heads. After removing the roof, side walls and floor of the nasal cavity, there were no recognisable landmarks of the organ, which is hidden under a triangular elevation in the mucosa (Fig. 2C). A transverse section confirmed the identity of the organ (Fig. 2B). The opening of the VNO was not visualised macroscopically, but the dorsal location of the organ suggested that it communicates directly to the nasal cavity, and not with the incisive duct (ID) as in some other non-rodent species. This was subsequently confirmed by serial histological sections. The incisive duct communicates the oral cavity with the nasal cavity through the incisive papilla. The presence of a wide palatine fissure in the skull allows this communication (Fig. 2E,F).

**Figure 3:**
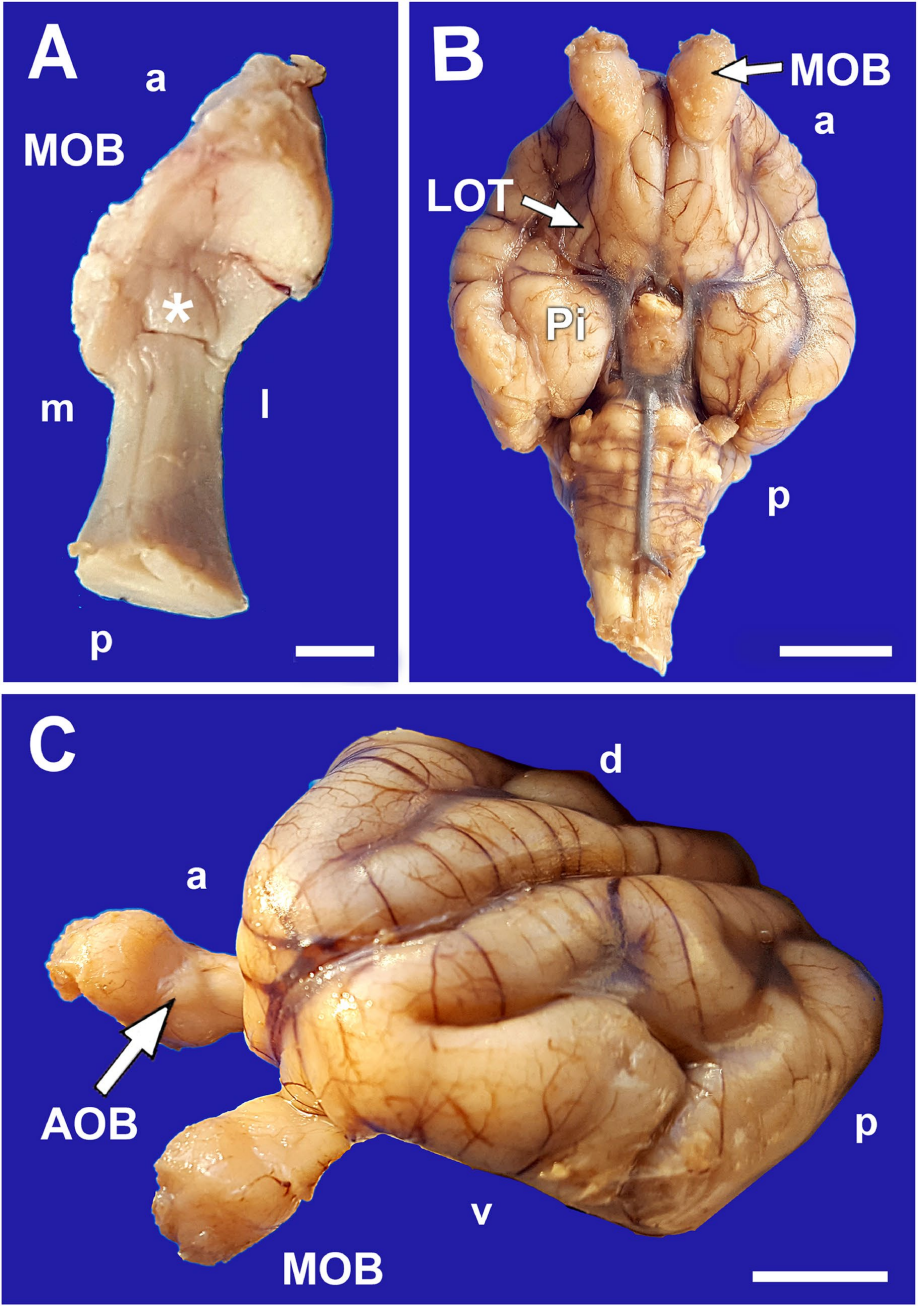
Olfactory bulbs of the neonate capybara. (**A**) Dorsal view of the right olfactory bulb showing the location of the AOB (asterisk). **(B)** Ventral view of the brain showing the topography of the olfactory pathway. MOB: Main olfactory bulb; LOT: Lateral olfactory tract; Pi: Piriform lobe. **(C)** Rostrolateral view of the brain where the MOB and the AOB (arrow) are differentiated. a: Anterior; p: posterior; d: dorsal; v: ventral. Scale bars: (**A**) 2.5 mm; (**B**,**C**) 1 cm.

The brain was removed to appreciate the degree of development and the topography of the olfactory portion, mainly formed by the OBs, the lateral olfactory tract and the piriform lobes (Fig. 3B). The main olfactory bulbs (MOB) were then sectioned at the peduncle level and the anterior frontal lobe was removed to visualise the AOB more easily. It corresponded to an oval elevation in the dorsocaudal area of the OB (Fig. 3A,C). This was delimited by blood vessels and the arrival of the vomeronasal nerve through its medial face was visualised.

The VNO in the newborn capybaras (P0) presents a capsule, a vomeronasal duct and the parenchyma.

**Figure 4:**
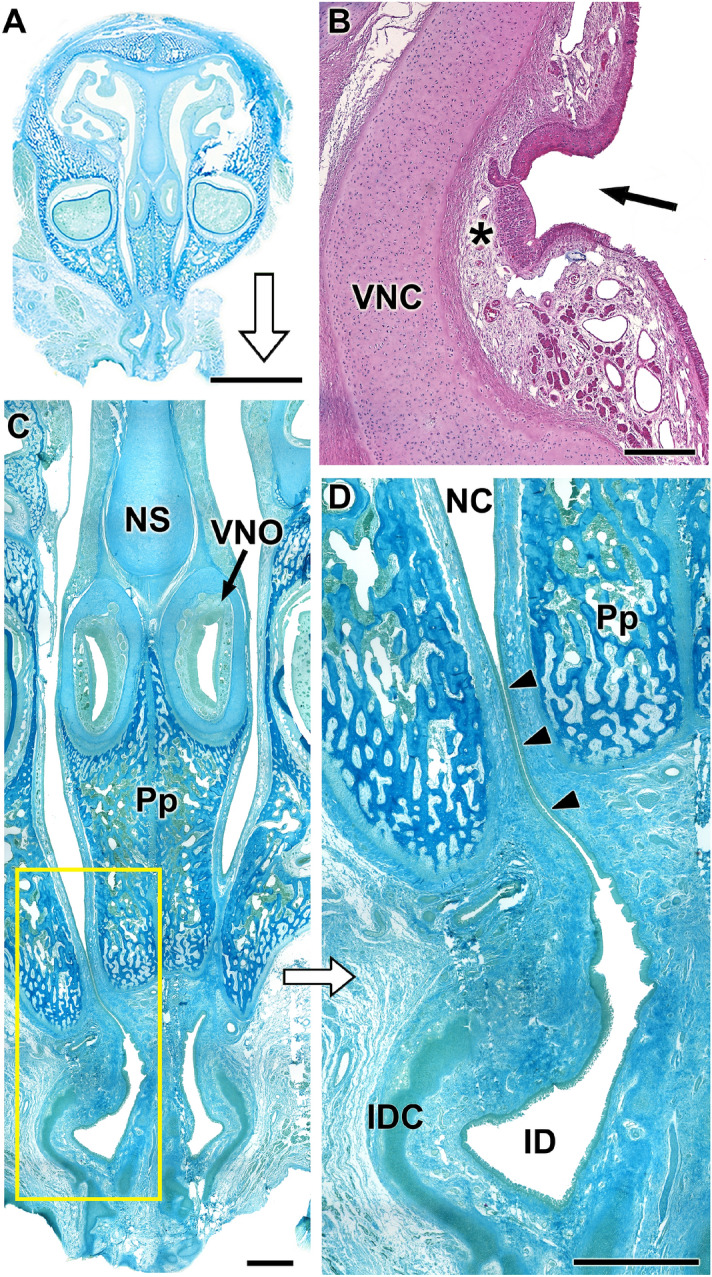
Histological images of the whole nasal cavity of the P0 capybara showing the topographical relationships of the VNO. (**A**,**C**,**D**) Successive magnifications showing the location of the VNO and the communication (arrowheads) established between the nasal and oral cavities through the incisive duct (ID). **(B)** Mouth of the vomeronasal duct in the nasal cavity (arrow). At this level, the receptor epithelium (asterisk) is already present. IDC: Incisive duct cartilage; NC: Nasal cavity; NS: Nasal septum; Pp: Palatal process of the incisive bone; VNC: Vomeronasal cartilage; VNO: Vomeronasal organ. Stainings: (**A**,**C**,**D**) Gallego’s trichrome; (**B**) Hematoxylin–Eosin. Scale bars: (**A**) 1 cm; (**B**) 250 µm; (**C**, **D**) 1 mm.

**Figure 5:**
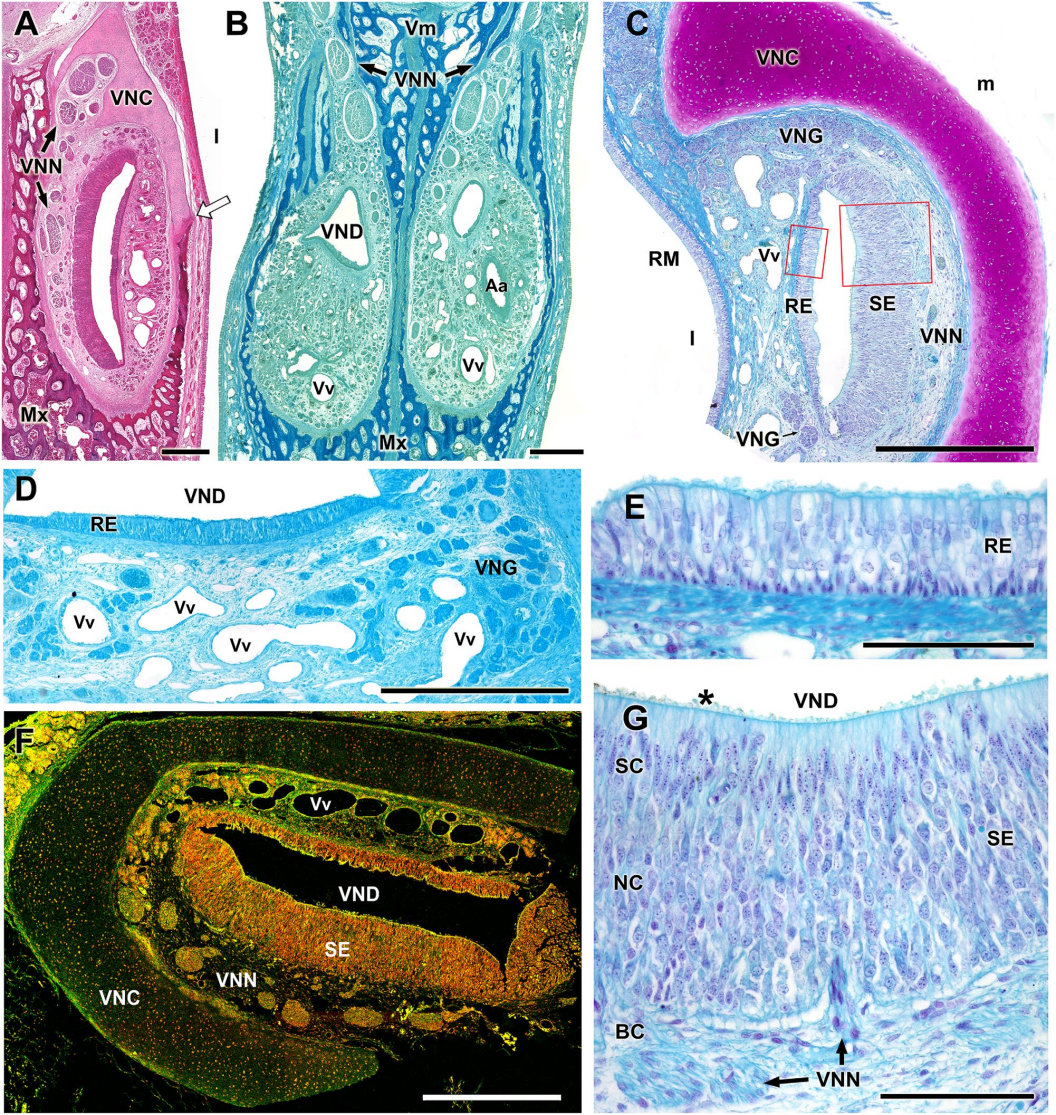
Histological sections of the capybara VNO showing its main components. (**A**,**B**) Transverse sections of the nasal septum exposing the nature of the vomeronasal capsule. **(A)** corresponds to the central level, where the cartilage is replaced ventrally by the dorsal projection of the maxillary bone (white arrow). **(B)** corresponds to a caudal level where the bone capsule fully encapsulates both VNOs. **(C)** Cross section of the VNO showing the main components in the parenchyma: Vomeronasal duct (VND) lined medially by sensory epithelium (SE) and laterally by respiratory epithelium (RE), vomeronasal glands (VNG), vomeronasal nerves (VNN), vomeronasal cartilage (VNC) and veins (Vv). Both epithelia, RE and SE, insets are magnified in figures **(E)** and **(G)** respectively. In the SE, the three cellular strata can be seen: SC, sustentacular cells; NC, neuroreceptor cells; and BC, basal cells. The microvilli (asterisk) contact with the lumen of the vomeronasal duct. **(D)** Enlargement of the dorsolateral area of the VNO showing the serous and AB + nature of the vomeronasal glands. **(F)** Study of the VNO irrigation by confocal microscopy showing veins along the lateral part of parenchyma. Elastin autofluorescence of a transversal section. Nuclear counterstaining with TO-PRO-3. Aa: Artery; Mx: Maxillary bone; MR: Respiratory mucosa of the nasal cavity: Vm: Vomer bone; l: lateral; m: medial. Stainings: (**A**) Hematoxylin–Eosin; (**B**,**C**,**E**,**G**) Gallego’s trichrome; (**D**) Alcian blue. Scale bars: (**A**–**D**) 500 µm; (**E**–**G**) 100 µm.

*Capsule* Well-developed, it completely envelopes the organ in its central part (Fig. 4A–C). Anteriorly, related to the opening of the vomeronasal duct, the lateral part of the capsule is absent (Fig. 4B). Caudally, it presents a dorsal groove for the exit of the nerves (Fig. 5B). The capsule is cartilaginous in nature but, in the caudal third it is progressively replaced from ventrodorsally by a bone capsule, which is noticeable in the adult skull (Fig. 2A,D). Ventrally, this bone capsule is an extension of the maxillary bone, and dorsally it is completed by the vomer bone (Fig. 5A,B).

*Vomeronasal duct* The duct is semicircular in section and is laterally covered by a respiratory epithelium and medially by a neuroreceptor epithelium, which in comparison has a significantly higher degree of development than the former (Fig. 5C–G). In the medial epithelium, three cell strata are distinguished: the sustentacular cell layer (SC) in the apical part, the neuroreceptor cell layer (NC) in the centre, and the basal cell layer (BC). The duct opens into the nasal cavity directly at its most anterior end through a small hole only visible in the histological series (Fig. 4B). Ventrally, the vomeronasal duct is related to the incisive duct (Fig. 4C). The ID ends in the incisive papilla, communicating in this way both the nasal and oral cavities. Additionally, the ID presents a cartilaginous envelope that prevents its collapse (Fig. 4D).

**Figure 6:**
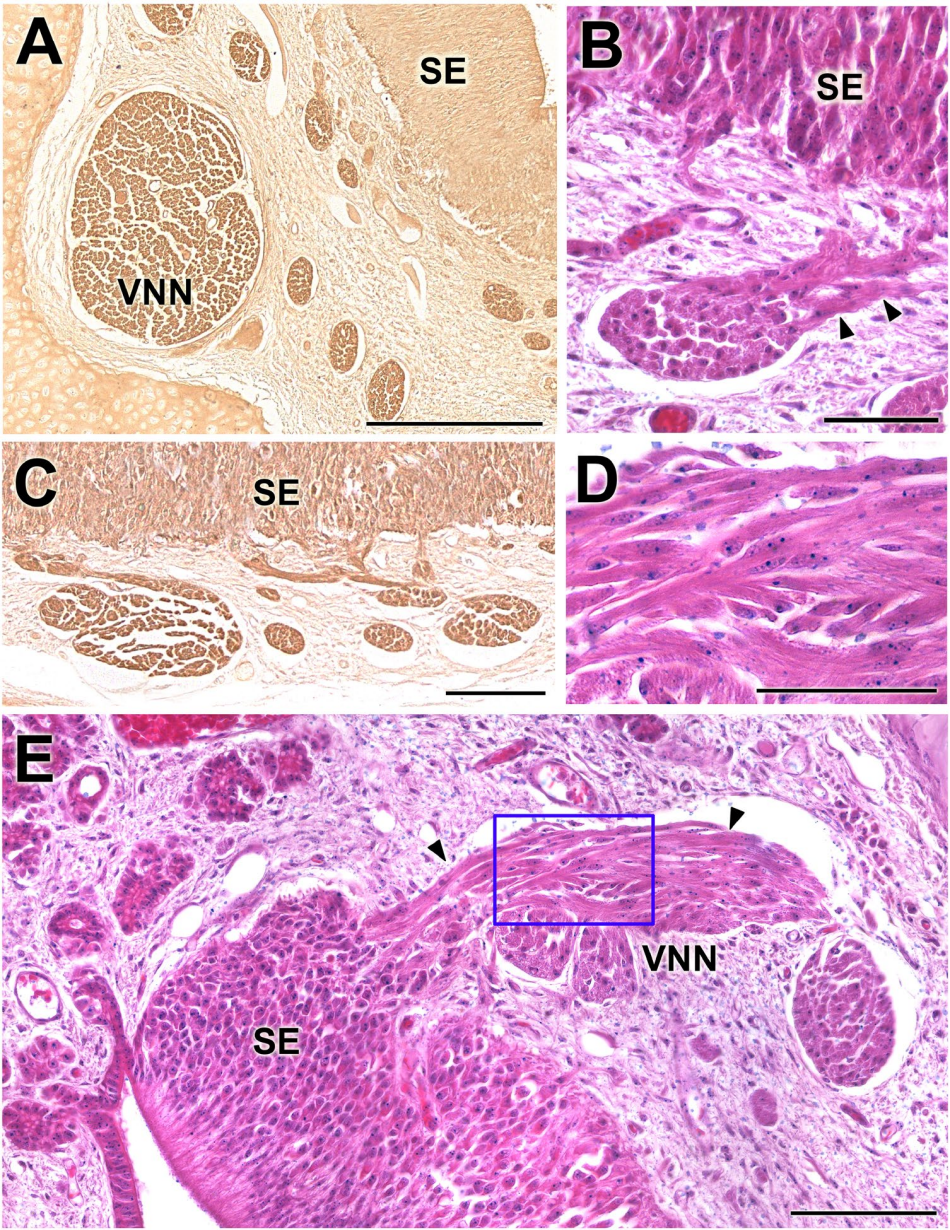
Histological study of the capybara vomeronasal nerves (VNN). (**A**,**C**) Large branches of the VNN in the dorsomedial (**A**) and medial (**C**) areas of the VNO immunostained by anti-GAP43. **(B**,**E)** Migratory stream of cells departing from the sensory epithelium (arrowheads). **(D)** Higher magnification of the inset showed in **E**. Stainings: (**B**,**D**,**E**) Hematoxylin–eosin. SE: sensory epithelium; VNN: Vomeronasal nerves. Scale bars: (**A**) 250 µm; (**B**,**D**) 50 µm; (**C**,**E**) 100 µm.

*Parenchyma* Its main components are vessels (mostly veins and sinuses), nerves, glands and loose connective tissue. The axons of the neuroreceptor cells converge, forming nerve fascicles (Fig. 6A) in the dorsomedial part of the parenchyma. They leave the parenchyma through the dorsal fissure of the capsule (Fig. 5B). At higher magnifications, individual nucleated cells can be seen leaving the sensory epithelium (SE) towards the nerve bundles, constituting a striking migratory stream (Fig. 6B–E). They are immunolabelled with anti-GAP-43 (Fig. 6A,C), whereas the labelling with anti-LHRH gave a negative result. The vasculature of the organ is poorly developed, presenting veins of moderate calibre and arteries of small calibre, which only become evident in the caudal zone, suggesting that the erectile character of the parenchyma is moderate (Fig. 5F). The vomeronasal glands, acinar, tubular or tubuloacinar, concentrated in the dorsal area (Fig. 5D). Caudally, the ends of the organs present a greater development of the glandular component (Fig. 5B). The morphology of the acini corresponds to the serous type, and its secretion is AB positive and very weakly PAS positive (Fig. 5A,D).

**Figure 7:**
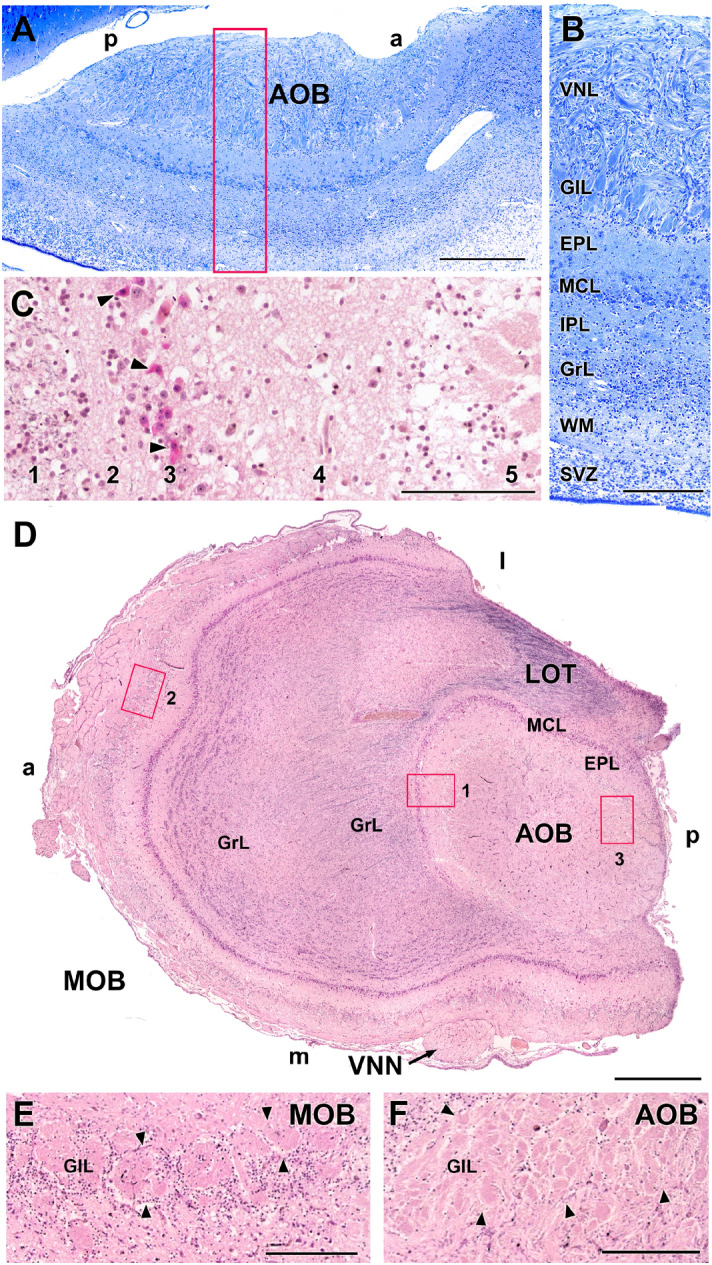
Capybara AOB microscopic study. (**A**) Sagittal section stained by Nissl. The laminar organisation is visible and showed at higher magnifications in **(B)**: Inset from (**A**) Vomeronasal nervous layer (VNL), glomerular layer (GlL), external plexiform layer (EPL), mitral cell layer (MCL), internal plexiform layer (IPL), granular cell layer (GCL), white matter (WM) and subventricular zone (SVZ). **(C)** Higher magnification of the inset 1 in (D). The Tolivia staining shows the polyhedric morphology of mitral cells (arrowheads). (1) GCL; (2) IPL; (3) MCL; (4) EPL; (5) GlL. **(D)** Horizontal section of the complete olfactory bulb stained with Tolivia to identify the convergence of myelinic fibres in the lateral olfactory tract (LOT). The differences in size and lamination of MOB and AOB and the arrival of the vomeronasal nerve (VNN) from the medial side of the olfactory bulb are noticeable. **(E)** Higher magnification of the inset 2 in (D) showing the GlL of the MOB **(F)** Higher magnification of the inset 3 in (**D**) showing the GlL of the AOB. a: Anterior; p: posterior; l: lateral; m: medial. Scale bars: (**A**) 500 µm; (**B**,**C**,**E**,**F**) 250 µm; (**D**) 1 mm.

The microscopic study of the AOB confirms its dorsocaudal location with respect to the MOB (Fig. 3C). Through sagittal sections, the characteristic laminar structure of the AOB is identified, differentiating the following strata: nervous, glomerular, external plexiform with a high degree of development, internal plexiform, a layer comprising numerous mitral cells between both plexiform layers named as mitral cell layer, and a deeper granular layer (Fig. 7A,B). The horizontal sections provide more information about the topography of the AOB and, in them, the arrival of the vomeronasal nerve can be seen through the medial surface of the OB (Fig. 7D). Tolivia staining allows the identification of abundant black myelinated fibres in the AOB granular stratum that converge in the LOT. In this staining, the mitral cells are stained red and show their polyhedric structure (Fig. 7C). The glomeruli in the AOB are smaller and less numerous than those in the MOB (Fig. 7E,F).

### Lectin histochemical and immunohistochemical study

**Figure 8:**
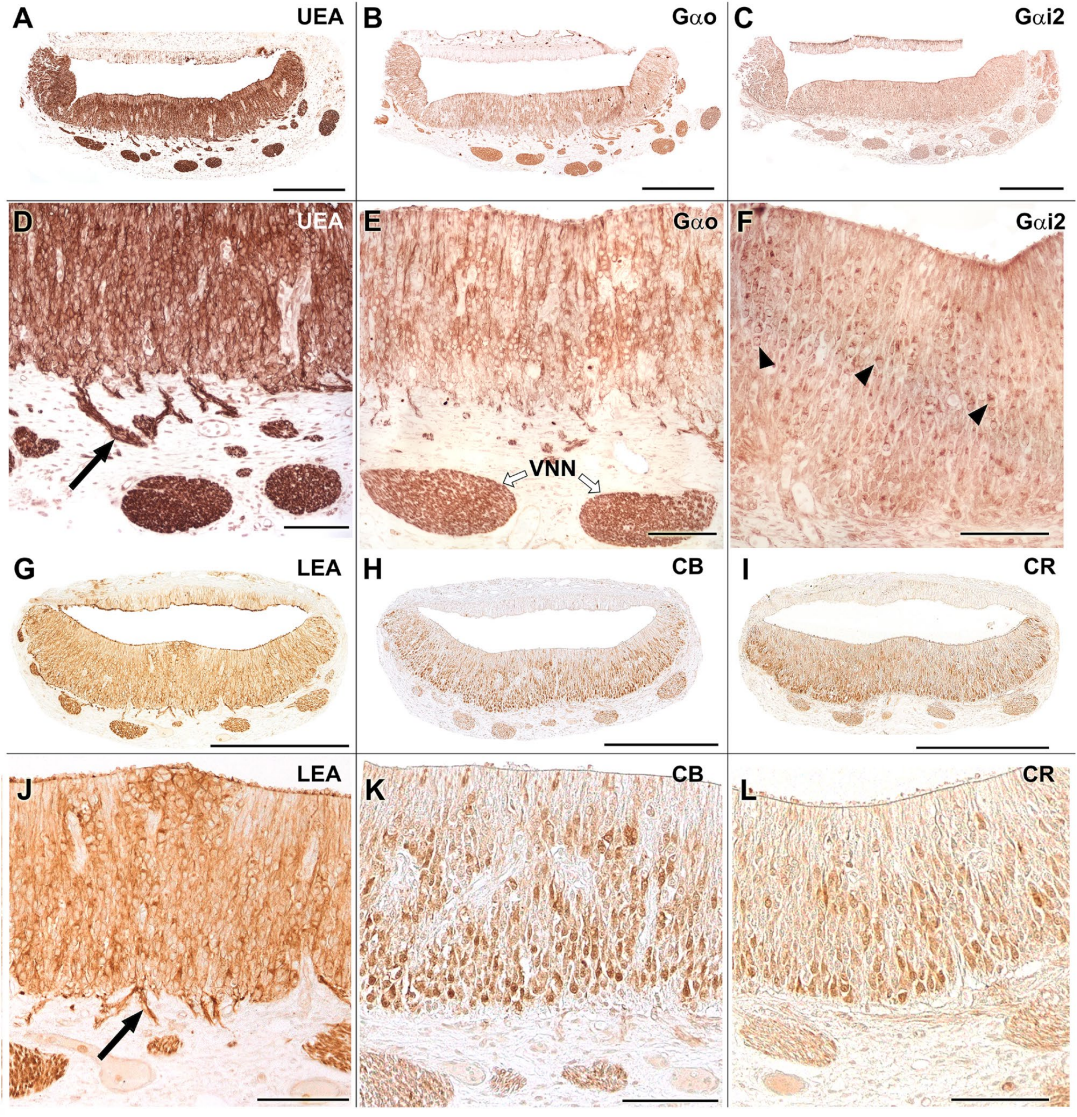
Capybara VNO histochemical and immunohistochemical labelling. (**A**,**D**) UEA lectin strongly marks both the entire sensory epithelium and vomeronasal nerves. It also allows the identification of the migratory current (arrow). **(B,E)** IHC labelling with anti-Gαo stains the vomeronasal nerves (white arrows) and produces a focally diffuse pattern in the neuroepithelium. **(C,F)** IHC labelling with anti-Gαi2 stains the nerve component and marks isolated receptor cells (arrowheads). **(G,J)** The LEA lectin produces a label similar to the UEA lectin, but not as thoroughly. A major part of the sensory epithelium and the vomeronasal nerves are marked. Migration is also identified (arrow). **(H,K)** Anti-Calbindin (CB) produces a cellular labelling distributed in the central and basal areas of the epithelium. **(I,L)** Anti-Calretinin (CR) produces a cellular labelling mainly concentrated in the basal area of the epithelium. Scale bars: (**A**–**C**, **G**–**I**) 500 µm; (**D**–**F**) 100 µm.

Both the UEA and the LEA lectins produce an intense label in the sensory epithelium (SE) of the VNO but are negative in the respiratory epithelium (RE) (Fig. 8A,G). The UEA lectin labels all of the cells, but the LEA lectin only labels a portion of the cell population (Fig. 8D,J). The vomeronasal nerves (VNNs) and the vomeronasal glands (VNGs) are also positive with both markers. Anti-Gαo and anti- Gαi2 produce immunolabelling in both the SE and in the VNN, (Fig. 8B,C,E,F). Calbindin and calretinin markers label the SE and the VNNs (Fig. 8H–I, K,L).

**Figure 9:**
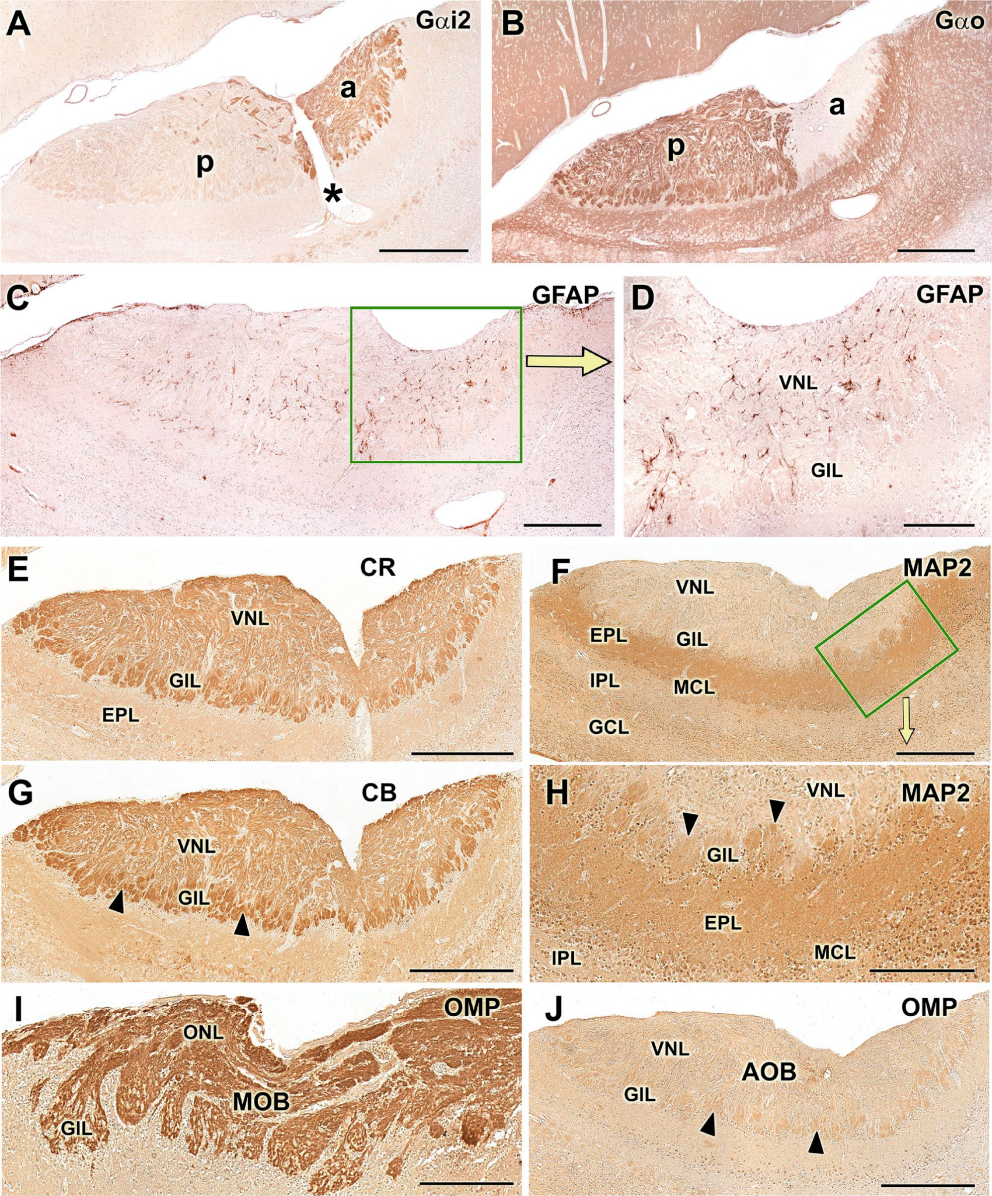
Immunohistochemical labelling in the capybara AOB. (**A**) The IHC labelling with anti-Gαi2 stains the nervous and glomerular layers of the anterior area of the AOB. **(B)** On the other hand, the anti-Gαo marks all of the nervous tissue except the anterior part of the AOB, resulting in a complementary expression pattern of both G proteins. **(C,D)** Marking with anti-GFAP produces a more prominent diffuse pattern in the nervous and glomerular part of the AOB. **(E,G)** In the AOB, both anti-Calretinin (CR) and anti-Calbindin (CB) produce a complete label more intense in the glomerular layer (arrowheads). **(F,H)** MAP2 labelling focuses on the external plexiform and in the glomerular layers (arrowheads). **(I,J)** The anti-OMP is immunopositive in the MOB, marking intensely the nervous and glomerular layers (GlL) whereas in the AOB the labelling is very faint, except in the GlL (arrowheads). a: Anterior; p: posterior. Scale bars: (**A**–**C**,**E**–**G** and **J**) 500 µm; (**D**,**H** and **I**) 250 µm.

In the AOB, the anti-Gαi2 and anti-Gαo immunohistochemical labellings followed an intense and complementary pattern. The first sharply marks the nervous and glomerular layers in the anterior area of the AOB. The anti-Gαo immunostains the same layers in the posterior area of the AOB as well as the rest of nervous tissue (Fig. 9A,B). Immunolabelling with anti-GFAP produces a more prominent diffuse pattern in the nervous and glomerular part of the AOB. It allows identification of the ensheathing glia of the nervous layer of the AOB, and a significant number of astrocytes (Fig. 9C,D). Calbindin and calretinin produce a complete labelling of the AOB without discriminating a zonation and specifically, CR revealed immunopositive mitral cell bodies (Fig. 9E,G). Anti-MAP-2 labelling focused on both the external plexiform and glomerular layers, but the latter is labelled with less intensity (Fig. 9F^,^H). The anti-OMP was strongly positive in the MOB, but in the AOB only marked the glomerular layer (Fig. 9I^,^J).

**Figure 10:**
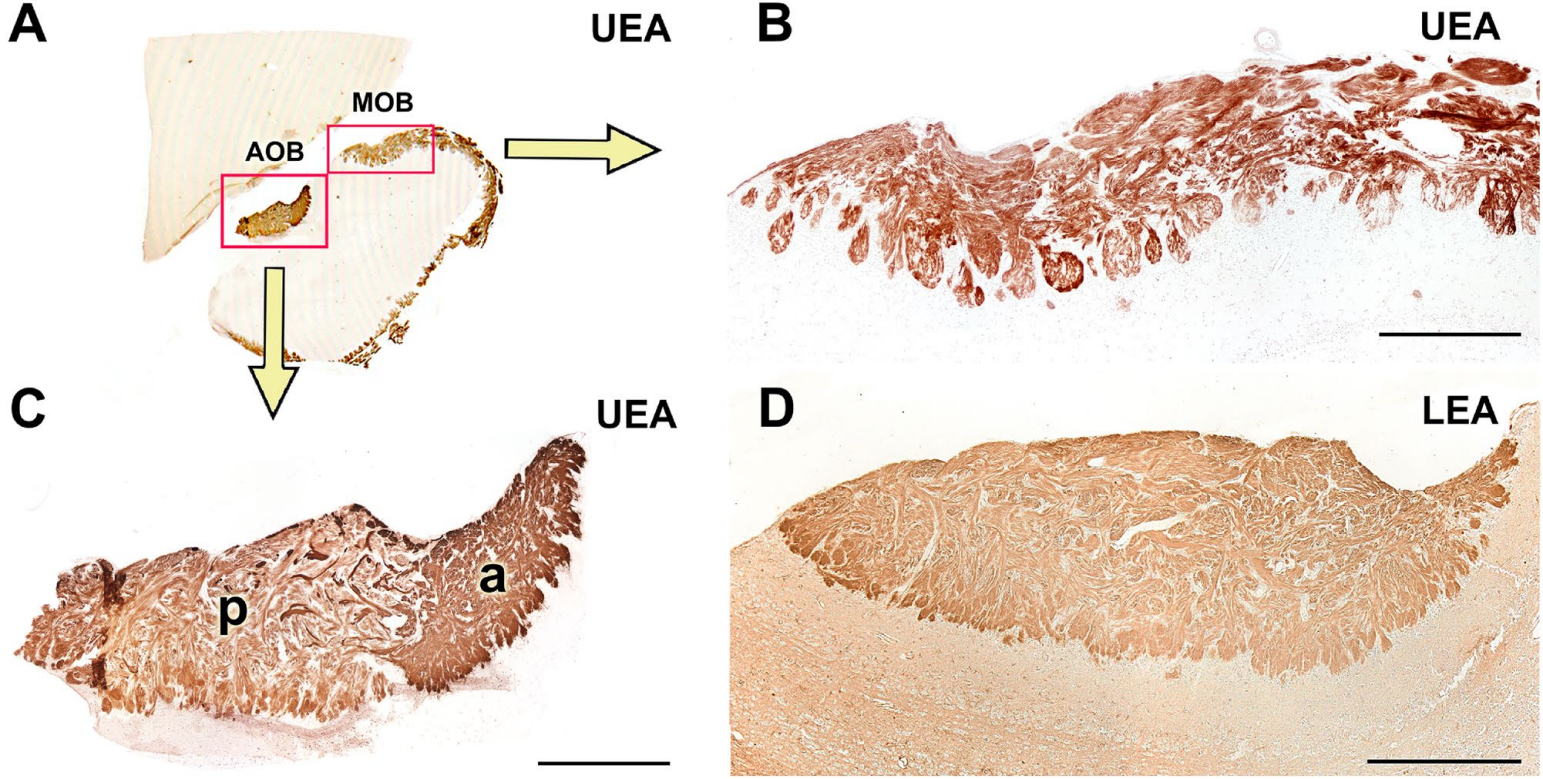
Lectin histochemical labelling in the capybara AOB. (**A**–**C**) UEA lectin is positive in both the nervous and glomerular strata of the entire olfactory bulb. In the AOB, it produces a slightly more intense labelling in the anterior area. **(D)** LEA lectin is positive in the entire AOB without differentiating zones. a: Anterior; p: posterior. Scale bars: (**B**) 250 µm; (**C**,**D**) 500 µm.

UEA lectin is positive in the nervous and glomerular strata of both the AOB and MOB. In the AOB, it produces a more intense marking in the anterior area (Fig. 10A–C). The LEA lectin completely marks the AOB without differentiating zones (Fig. 10D). Finally, the lectin BSI-B_4_ staining did not result in positive labelling, in either the VNO or the AOB.

## Discussion

There are significant structural, phylogenetic, and species-specific variations of the VNS between species^31^. Relative to the olfactory system, the VNS has at genomic level a greater diversity^4^. Comparative sequence analyses have demonstrated that MOE receptor gene sequences are well-conserved, whereas VNO receptors are associated with a wide variety of genes, suggesting a more dynamic evolution^32^. However, we must not forget that relationships between these systems reflect their common history of ecological adaptations during evolution^12^. In addition, pheromones are, by definition, specific for each species, and different species have different behavioural and reproductive strategies^33,34^. Given this huge diversity more morphofunctional studies of the VNS are needed to understand the basis of this genetic and behavioural multiplicity.

Currently, the most studied and representative VNS models among mammals are the mouse and the rat—species in which the VNS presents great morphofunctional development. However, these species comprise only a small fraction of the Rodentia family; therefore, we opted to study another rodent, the capybara, to determine whether the VNS of the mouse and rat are representative of the entire rodent family or whether significant differences exist in other species. Moreover, most studied rodents derive from laboratory strains, that omit natural conditions^35^. Studying a rodent species that has not undergone artificial selection by humans was another goal of this study. Finally, because the capybara is a precocial species, the use of newborn individuals allowed us to determine whether and how the VNS morphology had adapted to the requirements of a challenging environment, at birth.

Although valuable information on the expression of G proteins in the AOB of the adult capybara is available^23^, we have expanded these observations, addressing the main morphological and neurochemical features of both the AOB and the VNO. Our immunohistochemical study paid as well special attention to G proteins because new information on their perinatal expression could be relevant to understanding the role of the VNS at this stage of life. Adult capybara express the two families of vomeronasal receptors, V1R and V2R, which can be identified immunohistochemically by studying G protein expression^23^, as V1R neuroreceptor cells specifically possess the αi2 subunit of the G proteins in their sensory transduction chain, whereas V2R cells express the αo subunit.

As G proteins play a critical role in the pheromonal signal transduction chain, their immunopositivity along the neuroepithelium of the VNO, the VNN, and the nervous and glomerular layers of the P0 capybara AOB points to the existence of full maturity of the VNS in the newborn capybara (Fig. 8E,F). This fact is consistent with the high degree of maturity of the offspring at birth^36^. A comparable pattern of early prenatal development has been found in the VNS of other precocial mammals such as pigs^37^ and sheep^38^, although in neither case was the expression of G proteins in the VNS evaluated. The expression of G proteins observed in the AOB of the newborn capybara (Fig. 9A,B) establish a clear anteroposterior zonation, identical to that observed in the adult AOB^23^: the Gαi2 protein is expressed in the anterior area of the AOB and the Gαo protein in the posterior area. This zonation is also discriminated by the UEA lectin (Fig. 10C), which stained the anterior part more intensely. This pattern coincides with that observed in mice^15,39^. However, a later exhaustive study of UEA labelling in the mouse olfactory system^40^ could only identify such AOB zonation in 3 of the 16 mice studied. The authors concluded that these individual differences may be caused by the presence or absence of cues that activate receptor cells or may reflect the differences between wild and laboratory rodents. The presence of the UEA anteroposterior zonation in the capybara appears to confirm this theory regarding the differences between wild and laboratory animals. LEA lectin (Fig. 10D) stained equally both zones, being an useful marker of both olfactory systems.

Although the small number of available individuals available prevented the realization of a rigorous statistical study, all of our newborn specimens showed a caudal portion that was larger than the rostral portion, which demonstrated that starting at the perinatal stage, the capybara shows the bias toward a more prominent caudal AOB that was previously described in adult individuals by Suarez et al.^23,41^ These results, combined with the recent observations regarding the morphometrical parameters of the AOB, in two closely related degus species with contrasting social habits^20^, invite the hypothesis that some structural features of the AOB reflect the species lifestyle and arise during an early stage of the ontogeny.

We undertook further immunohistochemical and morphological studies that confirmed the functionality of the VNO and AOB at this early stage. In the VNO, the calcium-binding-proteins, calretinin and calbindin markers (Fig. 8K^,^L) stained the parenchymal nerve bundles and almost all of the sensory neuroepithelial cells, although they labelled different cell populations. Something similar happens in the shrew, where calretinin produces an intense labelling in almost all receptor cells and in the VNNs in prenatal individuals^42^. Both markers produced a similar labelling on the AOB, labelling the VNL and GlL layers, although the second one more intensely. In addition, calretinin produced a labelling of mitral cells as seen in prenatal studies in the shrew^42^ and adult hedgehog^43^.

The antibody to glial fibrillary acidic protein (Fig. 9C,D) labelled a significant number of astrocytes in the nerve and glomerular layers of the AOB. This fact is striking when compared with other species, since in most studies the astrocytic development at this early stage is hardly noticeable. In the case of the opossum, there is hardly any labelling with GFAP in a P0 individual^44^. Only studies in P0 mice show an astrocytic development close to that of the capybara, but it occurs in the intermediate superficial zone, which corresponds to a primitive stage of the internal plexiform layer. Therefore, the early labelling of astrocytes in P0 capybaras reinforces the idea that it presents a VNS with a high degree of maturity at birth.

Anti-microtubule-associated protein 2 (MAP-2) is an invaluable marker for the dendritic trees of mitral cells^46^. In the AOB of P0 capybara (Fig. 9F-H), the MAP-2 antibody produces diffuse labelling which is consistent with that seen in other species at birth such as rats, mice and opossum^44–48^. Anti-olfactory-marker-protein (OMP) produces an intense pattern of labelling in the nervous and glomerular layers of the MOB, but hardly produces labelling in the AOB, immunostaining only the glomerular layer weakly (Fig. 9I^,^J). It has been observed in other species that the nerve and glomerular layers of both bulbs are positive for the OMP marker after birth. However, it has also been seen that OMP marking has a considerable difference in the degree of intensity between AOB and MOB, being more intense in the case of MOB. This also happens in rodents such as the mouse or rat^49^ or in marsupials such as the opossum^50^.

In addition to this neurochemical findings, our study in the P0 capybara has provided evidence for certain morphological and immunohistochemical features unique to this species—for instance, the nature of the capsule that protects both VNOs, their dorsal location in the nasal cavity over the palatal process of the incisive bone, the high degree of morphological differentiation of the AOB at that early stage, and finally the presence of a migratory stream from the neuroepithelium of the VNO to the VNNs.

Macroscopically, the dorsal location of both VNOs is remarkable, as they rest on the palatal process of the incisive bone and not on the vomer bone, as is the case in most species (Figs. 1and 4). This topography is only comparable to that described in rabbits^51^, where the organs rest over a prominence of the vomer bone. Two patterns of communication of the vomeronasal duct with the outside have been described. In the first, the vomeronasal duct opens directly into the incisive duct, as is the case in most domestic mammals such as cows, dogs, horses and pigs^52^ as well as in lemurs^53^. But in the second, the VNO flows directly to the ventral meatus of the nasal cavity, in its rostral area. The latter is the case for all rodents^54^ and lagomorphs^51^ studied to date. Accordingly, in the capybara, both vomeronasal ducts open directly into the nasal cavity. Though in most species, the nature of the vomeronasal capsule is exclusively cartilaginous, there are species such as the mouse in which the capsule is formed by a thin bone sheet^39^ or doubly composed of cartilage and bone, as in the lagomorphs^51^. In the newborn capybara, we have observed a different pattern thus far unpublished in the literature,the capsule is initially cartilaginous, but from the central area of the organ it is progressively replaced from ventral to dorsal by a bone sheet that ends up completely enveloping the VNO in its caudal area (Figs.2A and 5A,B).

The development and maturity of the receptor neuroepithelium in newborn individuals (Fig. 5E) resembles the highly differentiated epithelium typical of another rodent, the rat^54,55^. The parenchyma presents moderate development of blood vessels (Fig. 5F). In this way, the capybara differs from other species such as cats^56^ or rabbits^51^, in which the development of the vascular pump is very sophisticated. The vomeronasal pump is the physiological mechanism by which pheromones dissolved in fluids are transported to the vomeronasal duct^57,58^.

The secretion of the vomeronasal glands to the duct of the organ plays a very important role in vomeronasal perireceptor processes^59,60^. The newborn capybara VNO shows a significant development of the glandular tissue, especially in the caudal end of the organ (Fig. 5B). However, in the central part of the duct, the presence of glands is more moderate. This pattern is analogous to that found in other rodents such as rats, guinea pigs^61^ and mice^62,63^. However, histochemically, their character in the capybara is both PAS + and AB + , which contrasts with that found in most rodents studied, which only express PAS + glandular secretion (rat and guinea pig^64^, mouse^62,65^, vole^66^, hamster^67^, chinchilla^16^). This notorious variation in gland characteristics within the same order may reflect an adaptation in capybaras to the aquatic nature of their habitat, which might require a specific pheromone-receptor interaction milieu.

All previous studies on the Rodentia vomeronasal glands (VNG) applying conventional histochemical techniques were done in adult specimens. There is less information regarding the prenatal and perinatal stages. We found in the newborn capybara that the VNGs are reactive to UEA-I, a lectin specific for mucous secretions^68^ and to LEA, which has been linked to the presence of sialic acid and galactosamine units in the glands^25,69^, and negative for the α-galactose-specific BSI-B_4_ lectin. These results agree with those found in newborn mice^39^, although in the latter case, the glandular development was less notable. Apart from rodents, precocial mammals show a remarkable prenatal development of the VNG, as Salazar et al.^38^ found LEA + glands in sheep foetus in the second trimester of pregnancy and in pig foetus in the fourth week of pregnancy^37,70^.

In the parenchyma of the newborn capybara, the VNNs are concentrated in the medial area of the organ, forming nervous fascicles that leave the parenchyma dorsally. An exceptional point about which there is no information in the literature is the profuse cellular migration that occurs from the sensory epithelium to the nerve fascicles (Fig. 6). This cell migration was positive for immunohistochemical labelling with anti-GAP-43 and histochemical labelling with both the UEA and LEA lectins, but it was negative when labelled with anti-LHRH; this indicates that this migration is not analogous to that described in rat and mouse prenatal foetuses, which originates from the vomeronasal part of the olfactory placode to the hypothalamus and is mostly comprised of immunopositive LHRH cells^71^. It is difficult to hypothesise about the significance of these cells since this is an unprecedented finding in both the olfactory and vomeronasal nerves. Although further studies should clarify the nature and fate of these cells, the immunopositivity for GAP-43 suggests to their neuronal nature.

With regard to the morphology of the AOB, our observations in the newborn capybara show that from birth, the macroscopic and microscopic organisation of the AOB are already defined, showing that the AOB of the capybara has a differentiated laminar pattern (Fig. 7), similar to that found in other rodents^39^, lagomorphs, marsupials and prosimians^72–74^. From the macroscopic point of view, it is remarkable that in the specimens studied by Suárez et al.^23^, the arrival of the VNN to the AOB is described as occurring from the lateral side of the bulb. Our dissections and histological series show that the arrival takes place through the medial side of the bulb (Fig. 7D), as happens in other rodents such as rats and mice and in most of the mammals studied, even belonging to other families^75,76^. However, as McCotter himself describes in the guinea pig^75^, a Hystricognathi rodent like the capybara constitutes an exception to this pattern, since in this species the arrival of the nerve takes place through the ventrolateral part of the bulb. Although we did not have access to adult individuals that could help us to elucidate this aspect, access to the Comparative Mammalian Brain Collections from the Universities of Wisconsin and Michigan State (https://brainmuseum.org/) allowed us to study a Nissl cross section of the adult capybara brain (Specimen 62-621, section 160). It clearly shows the arrival of the VNN to the AOB from the medial side of the left hemi-brain. Future studies should clarify this issue.

In summary, our observations show that after birth, the capybara VNS does possess: (1) A VNO that communicates directly with the nasal cavity, and indirectly with the oral cavity; (2) A VNO and an AOB that are morphologically similar to those of the adult; (3) Active secretory vomeronasal glands; (4) The same Gαo and Gαi2 sensitivity of the neurosensory epithelium, VNNs, and nervous and glomerular AOB layers as has been described in adult capybara; and (5) Almost all of the neurochemical markers employed show an activity typical of adult animals.

On the basis of these findings, the general conclusion may be drawn that the VNS of the capybara at birth is capable of establishing the same function as that of the adult animal. If we add to this our observations on the degree of differentiation of the AOB and the active cellular migration that occurs in the epithelium of the VNO, which is not described in any other mammalian species, we can conclude by considering the capybara as an excellent and promising model for the study of chemical communication in the first days of life.
